# Time-lagged genomic erosion and future environmental risks in a bird on the brink of extinction

**DOI:** 10.1098/rspb.2024.2480

**Published:** 2025-03-26

**Authors:** Xufen Liu, Ester Milesi, Claudia Fontsere, Hannah L. Owens, Robert Heinsohn, M. Thomas P. Gilbert, Ross Crates, David Nogués-Bravo, Hernán E. Morales

**Affiliations:** ^1^ Globe Institute, University of Copenhagen, Copenhagen, Denmark; ^2^ Informatics Department, University of Florida, Gainesville, FL, USA; ^3^ Fenner School of Environment and Society, Australian National University, Canberra, Australia; ^4^ University Museum, Norwegian University of Science and Technology, Trondheim, Trøndelag, Norway; ^5^ Department of Biology, Lund University, Lund, Sweden

**Keywords:** genomics, conservation, extinction, genetic diversity, genetic load, genomic erosion

## Abstract

Global biodiversity is rapidly declining due to habitat degradation and genomic erosion, highlighting the urgent need to monitor endangered species and their genetic health. Temporal genomics and ecological modelling offer finer resolution than single-time-point measurements, providing a comprehensive view of species’ recent and future trajectories. We investigated genomic erosion and environmental suitability in the critically endangered regent honeyeater (*Anthochaera phrygia*) by sequencing whole genomes of historical and modern specimens and building multi-temporal species distribution models (SDMs) across the last century. The species has declined from hundreds of thousands of individuals to fewer than 300 over the past 100 years. SDMs correctly predicted known patterns of local extinction in southeast Australia. Our demographic reconstructions revealed a gradual population decline from 2000 to 2500 years ago, sharply accelerating in the last 500 years due to climate variability and habitat loss. Despite this substantial demographic collapse, the regent honeyeater has lost only 9% of its genetic diversity, with no evidence of inbreeding or connectivity loss. Also, it exhibits higher diversity than many other threatened bird species. Forward-in-time genomic simulations indicate that this time lag between population decline and genetic diversity loss conceals the risk of ongoing genomic erosion into a future of rapidly degrading environmental suitability. Our work underscores the need for targeted conservation efforts and continuous genetic monitoring to prevent species extinction.

## 1. Introduction

Anthropogenic environmental change drives the ongoing climate crisis and ecological destruction, leading to global biodiversity loss [1,2]. A key consequence of the biodiversity crisis is genomic erosion, where the loss of intraspecific genetic diversity reduces populations' viability and adaptive potential, increasing extinction risk. This gradual process can be detrimental long term, even after successful conservation action [3–7]. Understanding and monitoring the extent and pace of genomic erosion alongside environmental changes is essential for effective conservation action. Genetic metrics such as diversity, population structure, inbreeding and effective population size (*N*_e_) are key components of the essential biodiversity variables (EBVs) [8]. These metrics are crucial for understanding how populations adapt and function, making their close monitoring valuable for conserving threatened species [8]. Genetic EBVs enable the reconstruction of demographic trends and provide key information for assessing extinction risk, especially when direct ecological data on species demography is unavailable. Although genetic EBVs are often estimated at a single point in time to guide conservation strategies [9–11], their effectiveness can be limited by the unique life history and past population dynamics of each species [12–14]. This is suggested by the weak and variable correlation between intraspecific genetic diversity and conservation status in the IUCN Red List [15]. This limitation is compounded by the frequent neglect of genetic diversity in conservation assessments and our limited understanding of how genomic erosion responds to population decline [5,16]. Therefore, comparing genetic EBVs within a species over time (i.e. ΔEBV, including temporal changes in population structure, genetic diversity, inbreeding and *N*_e_) can provide a more accurate picture of genomic erosion dynamics, particularly in species whose populations have recently declined [13]. Historical genomic data show that endangered species with recent population bottlenecks often experience genomic erosion, which includes lower genetic diversity, reduced *N*_e_ and an increase in harmful genetic variation, which exacerbates the population decline (e.g. [6,7,17]). Changes in genetic structure may also arise from demographic decline, worsening genomic erosion by intensifying inbreeding and genetic drift, or potentially leading to outbreeding depression [4]. This highlights the crucial role of temporal genomic datasets in identifying dynamics of genetic erosion over time to inform conservation efforts.

Temporal comparisons have shown that changes in genetic diversity lag behind changes in population size due to the genetic drift debt, an important but often overlooked aspect of risk assessments [18,19]. This time lag can be particularly prominent in highly mobile species with large population sizes, as it takes a long time for local extinctions and population bottlenecks to translate into genetic diversity loss [12,18–20]. To understand this delay and predict future trends in genomic erosion, it is important to include ecological data and records of past demographic events. Species distribution models (SDMs) are useful tools for measuring temporal trends in distributions and population dynamics [21]. Informed by historical environmental and occurrence data, SDMs can reconstruct the potential past distributions of species and range change dynamics, improving the assessment of drift debt in ΔEBVs without direct historical records. SDMs are also used to infer present and future suitable environments, which aids conservation planning by overcoming sampling biases and predicting possible range shifts under climate change [22,23]. However, since SDMs do not directly estimate intraspecific genetic diversity, relying solely on them can be less effective for assessing long-term species survival [22]. Therefore, conservation planning could benefit from integrating ecological insights from SDMs with temporal genomics for a comprehensive understanding of ΔEBVs.

The critically endangered regent honeyeater presents a strong case study for examining how environmental threats are exacerbated by the time-lag effects of genomic erosion in endangered species. This nomadic species inhabits the fertile forests and woodlands of southeastern Australia, relying primarily on eucalyptus blossoms and invertebrates for food, as well as eucalyptus forests for breeding [24]. Over recent decades, the regent honeyeater has dramatically declined. Historically, its range extended from Adelaide to northern Queensland [25]. Late-19th-century reports described flocks ‘in thousands’, with aggregations of over 80 individuals until the 1970s [25]. European settlement and colonization in the late 18th century led to widespread forest clearance, drastically reducing regent honeyeater populations, with the South Australian population going extinct in the 1980s. Fewer than 250 mature individuals are left in the wild [24]. Despite an ongoing extensive conservation plan including zoo-breeding for reintroduction [26], management of competitors and protection of nests from predation [27], more effective conservation efforts based on monitoring data are needed to save the species from extinction [28].

We sequenced whole genomes of 24 museum-preserved (>100-year-old) and 20 modern (from 2011 to 2016) regent honeyeaters to investigate four temporal ΔEBVs (population structure, genetic diversity, inbreeding and *N*_e_), and resolve the temporal dynamics of genomic erosion in this widely dispersed, highly mobile species. We also built a multi-temporal SDM to infer past and future trends in environmental suitability across the species range to assess the potential impact of environmental change. Finally, we used genomic individual-based simulations to illustrate how time-lagged genomic erosion obscures patterns of genomic erosion after population collapse, demonstrating the value of integrating temporal genetic EBVs and ecological modelling in conservation assessments.

## 2. Methods

### (a) Sampling and sequencing

**Figure 1.**
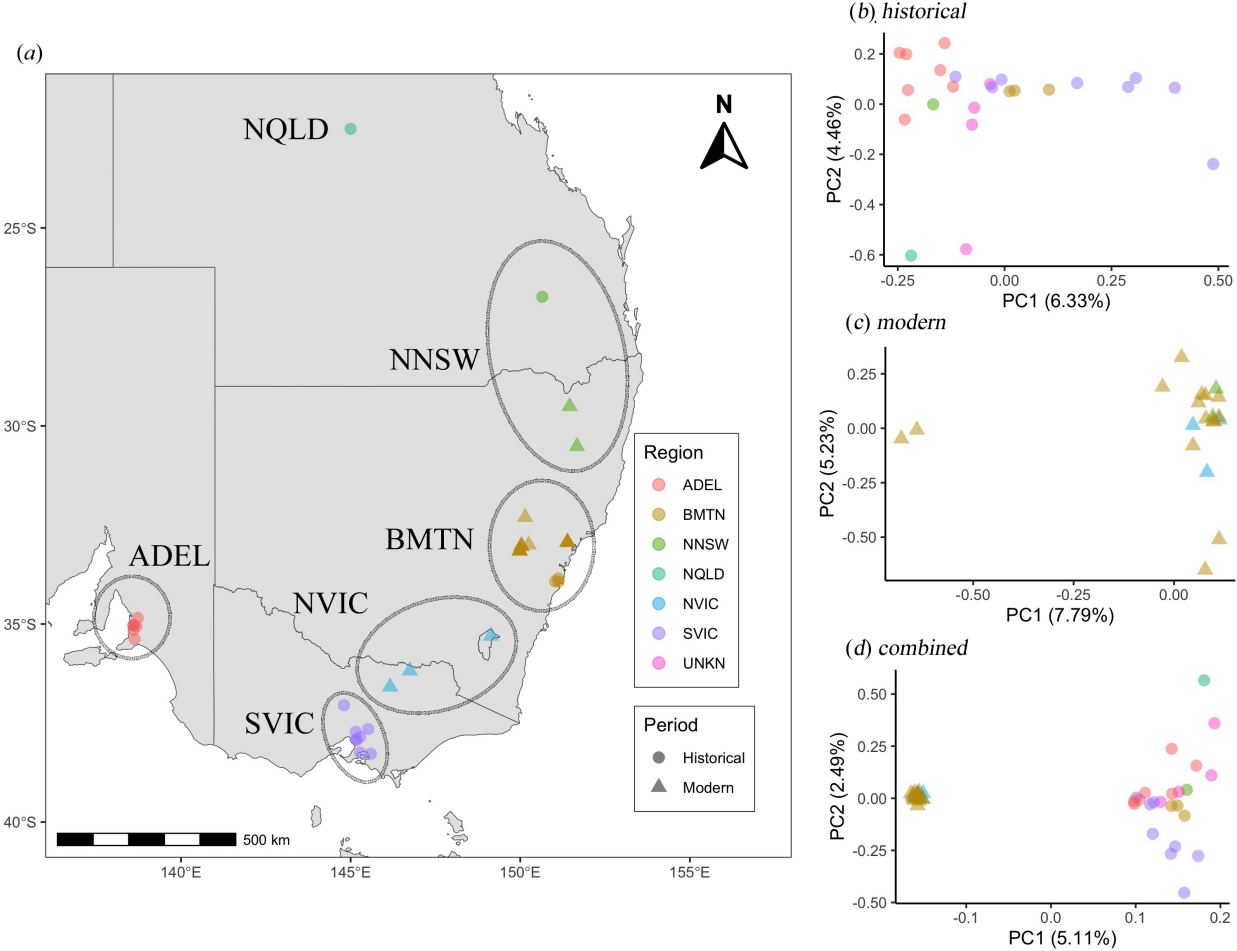
Geographical location and genetic structure of historical and modern regent honeyeater samples. (*a*) Approximate location of historical (triangles) and modern (circles) samples across different geographic regions (colours). (*b–d*) Principal component analysis of (*b*) historical (2 715 045 sites), (*c*) modern (2 009 752 sites) and (*d*) all samples (2 139 730 sites). Abbreviations of the regions are northern New South Wales (NNSW), greater Blue Mountains (BMTN), northern Victoria (NVIC) and southern Victoria (SVIC). Sampling periods are historical (1879−1900) and modern (2011−2016).

We re-sequenced the genomes of 44 *Anthochaera phrygia* specimens across most of the known extant and historical range of the species, including 20 ‘modern’ individuals from 2011 to 2016 and 24 ‘historical’ individuals before 1919 (figure 1*a*; electronic supplementary material, table S1). Among the geographic regions, the greater Blue Mountains (BMTN) sustain the largest remaining wild regent honeyeater population, with the highest nesting success [29,30].

Modern blood samples were extracted with DNeasy Blood and Tissue Kit (Qiagen, Hilden, Germany) and submitted to BGI-Europe for library building and paired-end 150 bp sequencing using a DNBSEQ-G400. Historical toe-pad samples were extracted following Gilbert *et al*. [31] in ultra-clean laboratories for ancient DNA at the University of Copenhagen. Genomic libraries were built following Kapp *et al*. [32] as modified for the BGISEQ sequencing platform [33] with library adapters designed for the BGISEQ sequencing Platform [34] and submitted to BGI-Europe for pair-end 100 bp sequencing. We checked the sequencing quality of all samples using FastQC v0.11.9 [35].

### (b) Read alignment and data preparation

We used PALEOMIX [36] to clean and map raw sequencing reads using bwa-mem [37] for modern and bwa-aln [38] for historical samples. Read optical duplicates were removed with Picard tools v.2.26.2 and sequences realigned around InDels with GATK v3.8.1 [39,40]. DNA damage was estimated with mapDamage 2.0 [41]. We mapped the reads to the chromosome-level reference genome of the helmeted honeyeater (*Lichenostomus melanops cassidix*) [42], a closely related species hat diverged from *A. phrygia* approximately 14 million years ago (MYA) [43]. Mapping to this sister species reduces reference biases as the helmeted honeyeater is a phylogenetically equidistant outgroup for both modern and historical samples. We used ANGSD v0.940 and samtools to estimate the depth of coverage (DOC) and its distribution [44,45] resulting in a genome-wide coverage depth range of 7.99 to 10.07 (mean = 9.17, s.d. = 0.51) for modern and 2.13 to 7.67 (mean = 4.84, s.d. = 1.68) for historical samples (electronic supplementary material, table S1). For all analyses, only autosomes were included and we confirmed that our sampling did not contain closely related individuals by assessing pairwise relatedness with NgsRelate V2 [46].

Historical DNA is subject to postmortem DNA damage and contamination, resulting in short sequencing reads, low endogenous content, higher error rate and an overall low depth of coverage. To counteract these potential biases, we used dedicated software for low-coverage samples, ANGSD v0.940 [44], to estimate genotype likelihoods and avoid directly calling genotypes. We also obtained a list of 640 492 803 high-quality sites with set of strict filters across all ANGSD methods. We removed transitions to account for DNA damage (-rmTrans 1), reads with multiple best mapping hits (-uniqueOnly 1), multiallelic sites (-skipTriallelic 1) and not primary reads (-remove_bads 1), reads with a quality score lower than 20 (-minQ), and mapping quality lower 20 (-minMapQ), sites supported by depth of coverage lower than four (-setMinDepth) and sites that were not present in at least two individuals (-minInd).

### (c) Population structure and demographic history

We explored patterns of population genetic structure by principal component analysis (PCA) using PCAngsd v1.01 and NGSadmix [44]. Two outlier samples were identified and removed from all further analyses (electronic supplementary material, figure S1). We reconstructed the historical and recent trajectory of effective population size (*N*_e_) using two methods with different temporal resolutions. We used Stairwayplot v2.1 [47] to reconstruct historical *N*_e_ between 10 000 and 300 years before the present, with the site frequency spectrum (ANGSD with the modern population) and a mutation rate of 4.6 × 10^–9^ following Smeds *et al*. [48]. Next, we used GONE [49] to reconstruct historical *N*_e_ over the last 300 years by calling high-quality single nucleotide polymorphisms (SNPs) with ANGSD (*p*-value threshold 1 × 10^−6^) and filtered sites that deviated from Hardy–Weinberg equilibrium. We used a jackknife approach to resample individuals and a maximum of 50 000 SNPs per scaffold in each iteration. A generation time of 3.4 years was used in both analyses [50].

### (d) Genetic diversity and inbreeding

We estimated per-individual genome-wide heterozygosity (H) with a site frequency spectrum from realSFS in ANGSD v0.940 [44]. To compare the level of genetic diversity of the regent honeyeater to that of other bird species, we downloaded the genomes of 33 bird species distributed across the avian tree of life, representing various conservation status classifications (electronic supplementary material, table S2). Mapping was done with bwa 0.7.17 mem [38] for short reads and pbmm2 v1.13.1 for long reads [51] with default settings. We downsampled bam files with samtools view -s [52] to the 10× coverage of our modern samples to avoid biases.

We measured per-individual inbreeding coefficients with ngsF v1.2.0 [53] with five iterative runs. Next, we quantified runs of homozygosity (ROH) with ROHan 20230903 [54] and Plink 1.9 [55]. Plink requires calling SNP and thus was only run for modern individuals with parameters: --homozyg-kb 10, --homozyg-gap 100, --homozyg-snp 50, --homozyg-density 50--homozyg-window-snp 30, --homozyg-window-threshold 0.05, --homozyg-window-missing 5, --homozyg-window-het 5. A different combination of plink parameters were tested, generating the same results. ROHan was run on both historical and modern individuals, by incorporating the signal of DNA damage with estimateDamage.pl., and with parameters of 500 kbp window size and -rohmu of 2 × 10^–5^. We estimated coalescence time of ROHs with the equation *t* = (100/(L × cM))/2, where *t* = time in generations ago, L = ROH length in Mb and cM = recombination rate of 1.86−1.71 cM Mb⁻¹ [42].

### (e) Species distribution modelling

We used a multi-temporal calibration approach to capture range dynamics under a changing environment [56–58]. This SDM method enhances prediction accuracy by integrating data across different time periods instead of relying on a single time point [56,59]. We used presence data from 1901 to 2015 obtained from the Global Biodiversity Information Facility (www.gbif.org; electronic supplementary material, figure S2). Georeferenced presence records for each year were transformed into binary spatial data, with presence grids (P) representing areas with one or more species records and pseudo-absence grids (PA) representing areas where the species was never recorded.

To avoid biases caused by the varying availability of presence data for the early 20th century, subsets with an equal number of presences for each decade were created (electronic supplementary material, figure S2b). These subsets were used to build 10 independent replicate models using a generalized additive model (GAM) algorithm with 70% training and 30% test data. We used GAMs due to their predictive performance and interpretability, allowing us to model species distributions while providing clear insights into relationships between predictor variables and species occurrence. Models, projections and ensemble procedures were carried out using BIOMOD2, a modelling package implemented in R [60,61].

Climatic and land-use variables (electronic supplementary material, table S3) were selected based on their biological relevance for the species characteristics in the available literature [29,62,63]. Mean temperature and precipitation were selected (Bio 1, Bio 12) for average climatic conditions and extreme conditions (Bio 5, Bio 6) were considered to account for low temperatures in alpine regions. Six land-use categories were considered: (i) cropland; grazing land, divided in (ii) managed pasture and (iii) rangeland, (iv) forested primary land, (v) forested secondary land and (vi) urban environment. Climatic data were obtained from the CHELSA dataset [64]. Yearly data were only available for years 1901−2015, while future data were provided for three discrete periods: 2011−2040, 2041−2070 and 2071−2100. Bioclimatic variables used as variables for years 1901−2015 were derived using the function ‘biovars’ included in the R package ‘dismo’ [65]. Historical and future land-use data were obtained from the Land-Use Harmonization project [65] that provides yearly information on the portion of occupied grid-cell (ranging from 0 to 1) for each of the land-use categories.

The model was projected into the future over three periods (2015−2040, 2041−2070 and 2071−2100) considering three climate change scenarios, i.e. shared socioeconomic pathways (SSPs): SSP1-2.6 (CO_2_ emissions cut to net zero around 2075), SSP3-7.0 (CO_2_ emissions double by 2100) and SSP5-8.5 (CO_2_ emissions triple by 2075). A multivariate environmental similarity surface (MESS) analysis was conducted to identify potential model extrapolation issues. MESS was needed because the focus was to transfer model predictions to a different time period [66]. For more details on the entire modelling procedure, see the supplementary methods in the electronic supplementary material.

### (f) Genomic individual-based simulations

We used simulations of neutral and deleterious variation to investigate the effect of demographic history on the dynamics of genomic erosion of regent honeyeaters facing sustained population decline. Individual-based, forward-in-time simulations were performed in SLIM 3.6 [67] using a non-Wright–Fisher model with random mating and overlapping generations for faster simulations. We simulated a genomic region modelled after chromosome 23 of the collared flycatcher genome (12.3 Mb) [68], incorporating realistic exon, intron and intergenic region positions, as well as an underlying recombination map, thereby accurately representing linkage dynamics. We also simulated an exome architecture of 30 000 genes of 400 bp each with a recombination rate of 1 × 10⁻⁴ per base-position per generation and no recombination within genes, to achieve realistic amounts of genome-wide deleterious variation. We simulated neutral and deleterious mutations at a relative proportion of 1 : 2.3, using selection coefficients from a custom distribution of fitness effects (DFE) as explained in the electronic supplementary information of Dussex *et al*. [69] and with a flat mutation rate of 2.30 × 10⁻^9^.

We simulated different ancestral population sizes; *N*_e_ = 12 000, 28 000 or 40 000, which undergoes a population decline for 15 generations (as observed in our demographic reconstructions; see §3). The total (meta)population *N*_e_ was equally divided into three populations exchanging migrants at 2% and 5% dispersal rates per generation, as described in Heinsohn *et al.* [28]. Migration rates remained constant throughout the simulation. We varied the intensity of the bottleneck to declines of *N*_e_ = 1000, 100 or 50, and sustained the population decline for 30 generations (100 years) to estimate the effect on future genomic erosion.

## 3. Results

### (a) Population structure and demographic history

The PCA shows a subtle clustering pattern for historical samples (figure 1*b*) and no clear structure for modern samples (figure 1*c*). Two modern samples from the BMTN region (ReHo19 and ReHo42) are differentiated along the PC1 axis, but it is unclear why as these samples are not outliers in terms of sampling origin, depth of coverage or mapping quality (electronic supplementary material, table S1), although we cannot rule out that these individuals might be long-distance migrants from another location. Across time points, the historical and modern samples are distinguished into two distinct clusters (figure 1*d*). While some of this signal could emerge from the differences in quality between historical and modern samples, our bioinformatic pipeline using only high-confidence sites, removing transitions and performing estimates from genotype likelihood, should minimize this effect. It should also be noted that the distribution of modern samples on the PCA is more contracted than that of the historical samples, suggesting that some loss of population structure has occurred over time. The lack of strong population structure in modern and historical samples was confirmed with NGSadmix (electronic supplementary material, figure S4), suggesting that despite some moderate differentiation between ADEL samples and other historical samples, all samples originate from a single admixed population, in agreement with previous genetic studies [70].

**Figure 2.**
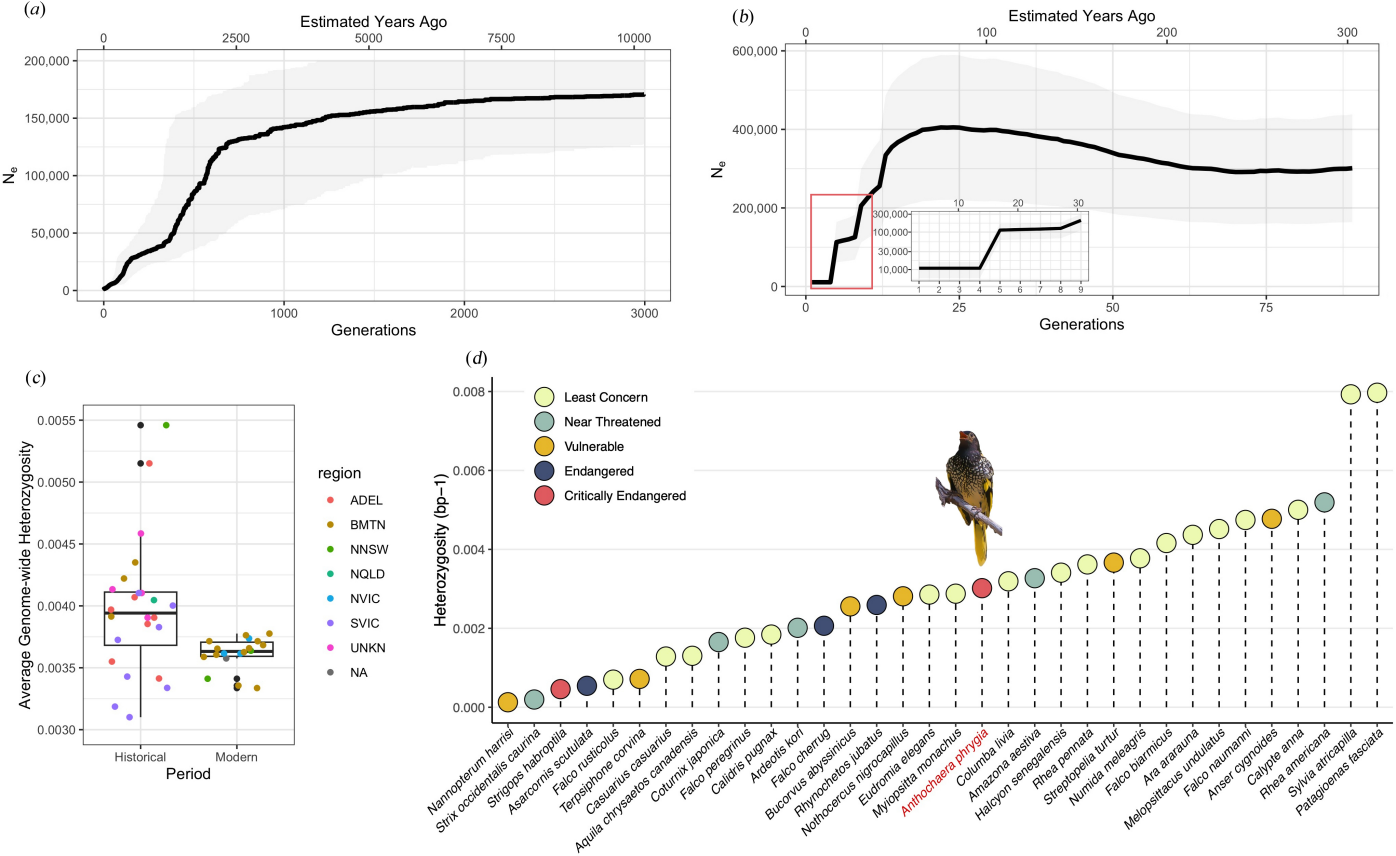
Demographic history and genetic diversity. (*a*) Effective population change from 10 000 years before the present (YBP) inferred by Stairwayplot. (*b*) Effective population change for the last 300 years by GONE. Thick black line: mean estimate; shaded colours: 95% confidence interval. The *y*-axis of the zoom-in figure of the last 10 generations is in log-10 scale. (*c*) Average genome-wide heterozygosity distribution of historical (1879−1900) and modern (2011−2016) individuals. Coloured dots: each sample with colours denoting its region (see figure 1*a* for abbreviations). (*d*) Average genome-wide heterozygosity across bird species processed with the same pipeline, ordered from low to high. Colour-filled circles represent their IUCN Red List conservation status (electronic supplementary material, table S2). The regent honeyeater (*Anthochaera phrygia*) is highlighted in red and with an icon. Average heterozygosity of modern samples is used.

Stairwayplot reconstruction of historical effective population size (*N*_e_) over the last 10 000 years indicates a decline beginning around 2500 to 2000 years ago. This decline accelerated in the last 500 years, with a more recent, steep drop in *N*_e_ starting approximately 70 years ago, corresponding with the observed contraction of suitable habitats in southern Australia (figure 2*a*). The demographic reconstruction of the last 300 years using GONE supports this pattern of decline (figure 2*b*), showing a population crash with *N*_e_ dropping from 400 000 to approximately 13 800 during this period, consistent with the documented decline in the number of recorded observations over the previous century. While the absolute *N*_e_ values differ between methods, as expected from operating on different genetic signals and assumptions, both models indicate a consistent downward trend in population size over time, reflecting the severe and ongoing population decline of the regent honeyeater.

### (b) Genetic diversity and inbreeding

Per-individual average heterozygosity in the modern population was found to be significantly lower than in the historical population, indicating a 9% loss of genetic diversity over time (Kruskal–Wallis test, *χ*^2^ = 4.4471, *p* = 0.035; figure 2*c*). This reduction is attributed to the population decline in the regent honeyeater, but is markedly lower than the >99% decline in population size, highlighting the extent of drift debt and the time lag between demographic and genetic loss [19]. Historical samples exhibited a wider distribution of heterozygosity values than modern samples, with the highest heterozygosity recorded in historical BMTN samples. This suggests that the genetic diversity within the species was once more variable among individuals and locations but has since become more uniform and lower. Despite this significant loss of genetic diversity, the regent honeyeater still retains more genetic diversity than 19 of 35 bird species included in a comparative analysis (figure 2*d*), even surpassing several non-threatened species on the IUCN Red List.

Inbreeding levels were found to be low in both historical and modern populations. The per-individual inbreeding coefficient in the modern population was very low, with a mean of 0.0069 and a s.d. of 0.00026, indicating minimal inbreeding. Furthermore, the analysis of ROH using ROHan revealed no evidence of ROH greater than 0.5 Mb in either the historical or modern samples, consistent with no evidence of recent inbreeding. We identified shorter ROH in the modern samples with plink (mean = 22.60 kb; s.d. = 22.11 kb; electronic supplementary material, tables S45–46). The presence of short ROH could be artefactual, or, if real, it could indicate an old signal of inbreeding dating back to 7506–6901 years ago, well before the recent population decline. These findings suggest that the regent honeyeater has avoided recent inbreeding and severe loss of genetic diversity.

### (c) Species distribution modelling

#### (i) Model evaluation

The performance of the model was robust, with the area under the curve of the receiver-operating characteristic (AUC) score averaging 0.877 (s.d. = 0.013) across models for the post-ensemble score (electronic supplementary material, table S5). An AUC value of 0.877 implies that there is a 87.7% chance that the model correctly classified presence and absence locations (a value of 0.5 would imply that the model is no better than random) [71]. The true skill statistic (TSS) averaged 0.791 (s.d. = 0.158), indicating that there is a 79.1% chance that the model correctly classified presence and absence locations. The bioclimatic variable having the most influence on environmental suitability was annual precipitation (0.29 of 1.0) and the strongest predictor among land-use variables was the percentage of urban environment per grid (0.32 of 1.0) (electronic supplementary material, table S3). The MESS analysis identified areas where future environmental conditions differed from the training data (electronic supplementary material, figure S7). Since projections into conditions outside the training data range are prone to high uncertainty and potential extrapolation errors, cells with negative MESS values were removed from mapped SDM projections.

#### (ii) Environmental suitability over time

**Figure 3.**
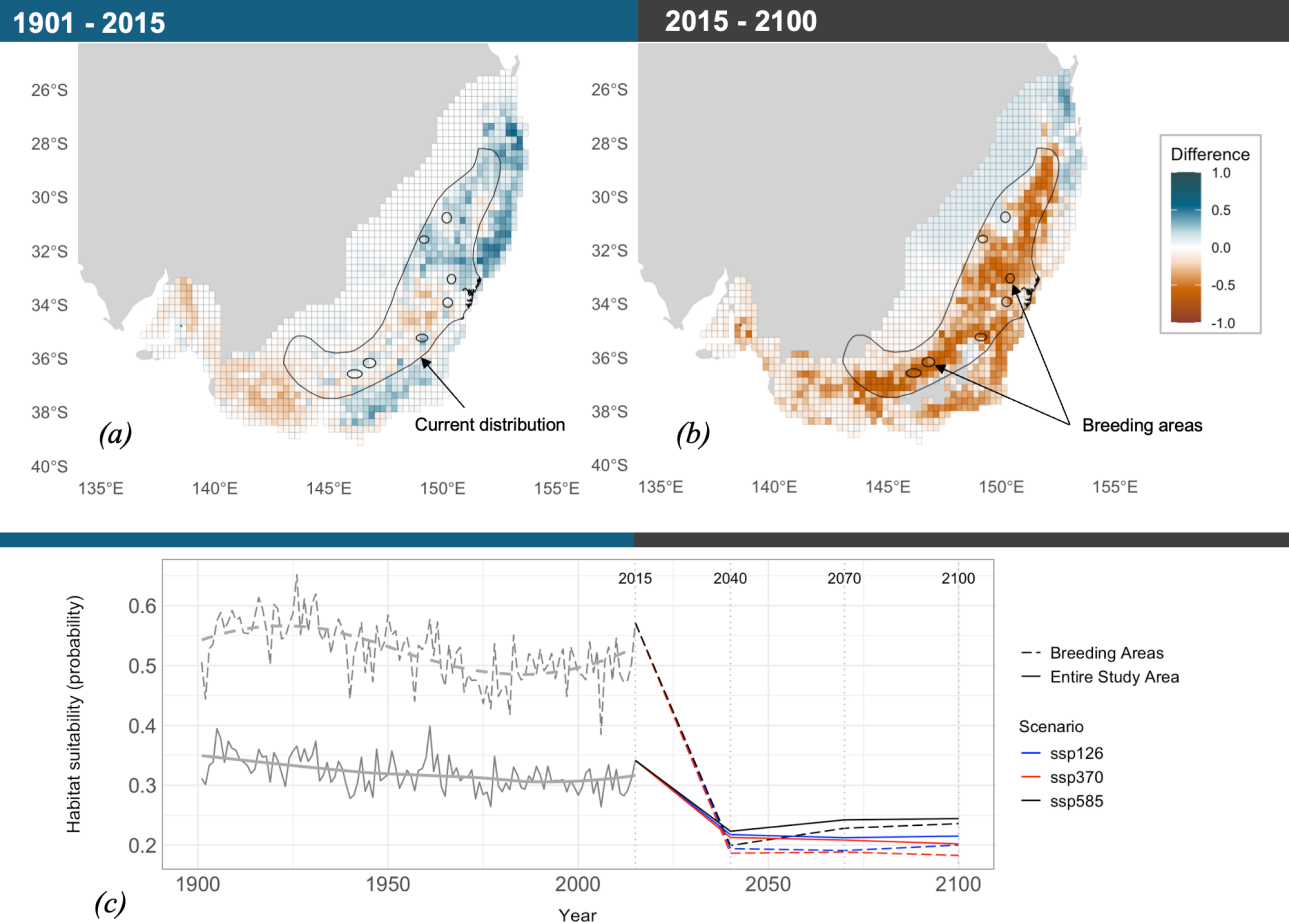
Multi-temporal species distribution model. (*a*) Probability of environmental suitability difference between 1901 and 2015. (*b*) Probability of environmental suitability difference between 2015 and 2100 for scenario SSP 3−7.0 (other future scenarios in electronic supplementary material, figure S8). Positive values indicate an increase in the probability of environmental suitability and negative values represent a decrease in the probability of environmental suitability relative to the present. (*c*) Habitat suitability trend over the full study period from 1901 to 2100. Probability values were extracted across the entire study area and for the breeding areas only. A loess curve was fitted to visualize nonlinear trends. Negative MESS values (see §3) were removed from the future projections. Polygons for the current distribution and breeding areas were obtained from Birdlife.org.

Over the past century (1901−2015), some areas experienced an increase in suitability over time, while others showed a decline (figure 3*a*). Notably, the southwestern regions exhibit a strong decline in suitability, highlighting the model’s ability to recover the known pattern of local extinction in areas where the species has not been recorded since the 1980s [26] due to a steady increase in cropland presence over this period [72].

Over the next century (2015−2100), the model predicted an overall decrease in mean suitability across the study area (figure 3*b*). The area of suitability loss mainly covers mountain areas, urban areas and cropland. An opposite trend was detected for a small coastal portion in the northeastern section of the study area, where suitability is expected to increase. Trends across breeding areas revealed a more severe loss over time, compared with the average across the whole study area (figure 3*c*), with the highest suitability loss (99% of the total) predicted between 2015 and 2040 (figure 3*c*).

### (d) Individual-based modelling

We simulated a demographic trajectory based on our reconstructions, testing ancestral populations of varying sizes and intensities of decline to estimate genomic metrics reflecting genetic diversity loss and accumulation of genetic load over time. We binned the simulated data into four time periods relative to 2016: (i) ‘historical’ (pre-1948), (ii) ‘modern’ (2009−2016), (iii) ‘10 years’ (2026−2033) and (iv) ‘100 years’ (2111−2118). Therefore, the historical category represents the pre-bottleneck ancestral population, the modern category represents the post-bottleneck population, and the 10 and 100 years categories represent the future projection of populations that have been bottlenecked for 25 and 50 generations, respectively.

**Figure 4.**
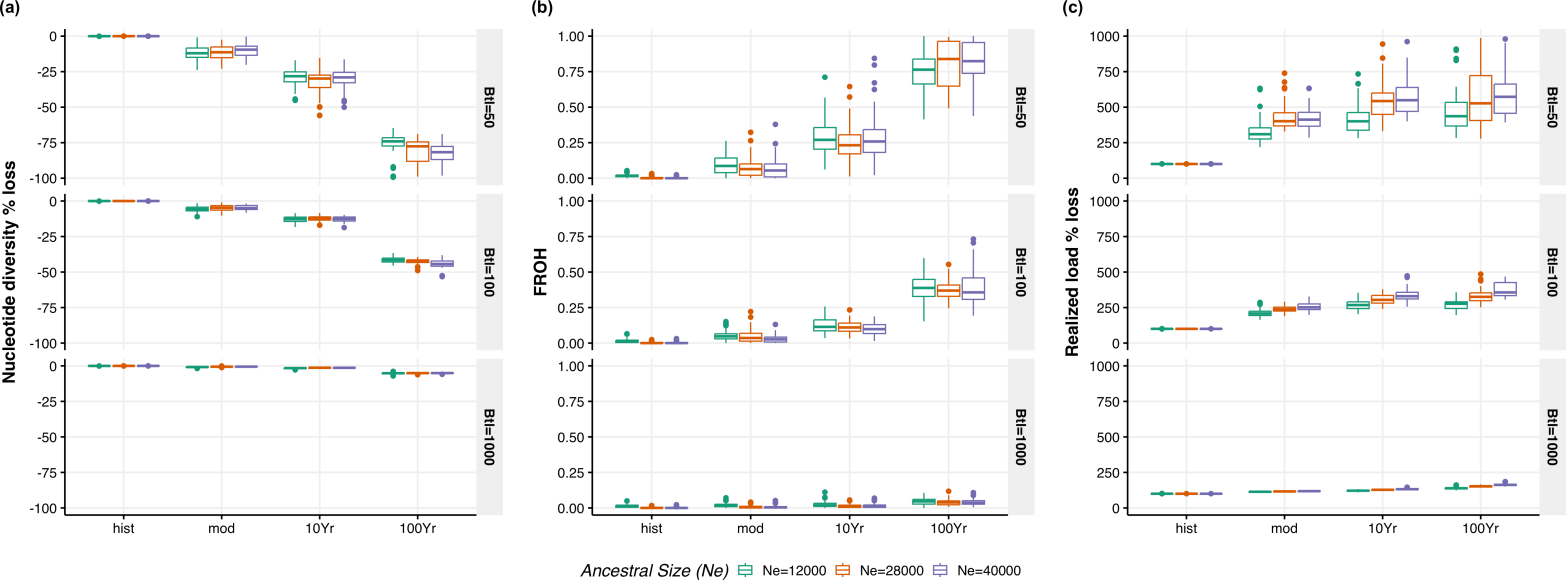
Simulated genomic erosion time lag after population decline from varying ancestral population sizes and bottleneck intensities. (*a*) Percentage loss of genetic diversity relative to the ancestral population. (*b*) Increase of the fraction of the genome in runs of homozygosity (FROH) relative to the ancestral population. (*c*) Percentage increase of the portion of the genetic load that expresses a negative fitness effect (realized load) relative to the ancestral population. The *x*-axis denotes the period of time for hist = historical, mod = modern, 10Yr = 10 years into the future and 100Yr = 100 years into the future. Colours represent the size of the ancestral metapopulation for *N*_e_ = 12 000, 28 000 or 40 000. The intensity of the bottleneck is shown for the different rows for *N*_e_ = 1000, 100 or 50.

The simulations show a time lag in genetic diversity loss relative to demographic decline due to genetic drift debt [19], with an initial modest loss that later accelerates in all scenarios (figure 4a). Compared with the empirical data (9% of genetic diversity loss), our simulations show that a genetic diversity loss of this magnitude requires a bottleneck smaller than *N*_e_ = 100 (simulated loss of 5.4%) and as low as *N*_e_ = 50 (simulated loss of 12%). This aligns with the modern regent honeyeater metapopulation’s lowest recorded census size of approximately 250–300 individuals [24,28]. Larger ancestral populations exhibit marginally lower levels of genetic diversity loss and fraction of the genome in runs of homozygosity (FROH) in the modern time point (figure 4*a*,*b*), probably due to the greater standing variation in these populations, which persists longer in bottlenecked populations. Contrary to this pattern, larger ancestral populations show a higher increase in the component of the genetic load that negatively affects fitness (i.e., the realized load) (figure 4c), probably due to their higher levels of ancestral deleterious variation, initially present as masked load in the pre-bottleneck populations [73] (electronic supplementary material, figure S9). Altogether, our simulations demonstrate that large ancestral populations, such as that of the regent honeyeater, experience a longer time lag in genetic diversity loss and a greater post-bottleneck accumulation of realized genetic load, making them more vulnerable to population declines.

Our simulations do not directly reflect the dynamics of actual regent honeyeater populations, as we simulated smaller ancestral sizes due to computational constraints and lack empirical estimates of genetic load for direct comparison. We thus emphasize that our simulations aim to illustrate the expected dynamics of genomic erosion under varying ancestral population sizes and bottleneck intensities, rather than the expected real effects in the wild population. However, our simulations use realistic values; the range of ancestral genetic load (3−4.5 lethal equivalents; electronic supplementary material, figure S9) approximates those reported across wild species (3–12 LEs) [74]. Given that the actual ancestral population size of regent honeyeaters was much larger, the genetic load in this species is likely to be larger than the one simulated here [73].

## 4. Discussion

By sequencing whole genomes from historical and modern regent honeyeater specimens and utilizing SDM, we gained insights into the genetic EBVs of the critically endangered regent honeyeater. This approach allowed us to assess genomic erosion and forecast potential declines in environmental suitability, highlighting the significant risks posed by deteriorating genetic health in the face of a rapidly degrading environment. Our findings underscore the value of incorporating temporal genomic and ecological data to comprehensively understand species’ conservation needs.

Despite severe population decline and habitat fragmentation, our analyses show that the regent honeyeater remains a single population with no evidence of inbreeding or genetic differentiation. However, we detected a decline in *N*_e_ and a modest but significant loss of genetic diversity, highlighting the genetic impact of the current population bottleneck. This complements earlier studies using lower-resolution markers [70,75], with our genome-wide analysis enabling more detailed demographic reconstructions that reveal a long-term decline from a large ancestral population that intensified in recent years.

Forward-in-time genomic simulations revealed a time lag between population decline and genetic diversity loss, posing a hidden risk of future genomic erosion. This risk is particularly acute for species with large ancestral populations that contain more recessive deleterious variation [5,6]. While the regent honeyeater retains relatively high genetic diversity compared with other endangered species, it faces ongoing risks from environmental degradation that could lead to rapid genomic erosion if conservation efforts are not intensified.

### (a) High mobility in a large, admixed population reduces the incidence of inbreeding

Regent honeyeaters have notably fewer and shorter ROH compared with other species with similar bottlenecks (e.g. black-faced spoonbill *Platalea minor* [76]). The lack of signals for inbreeding and genetic differentiation we detected is consistent with previous studies [29,75]. These short ROH date back to the end of deglaciation approximately 9000 years ago, well before European colonization and recent bottlenecks [77]. The low FROH and inbreeding coefficients in the declining regent honeyeater population may be due to the species's high mobility, allowing individuals to move efficiently, reducing the risk of inbreeding and population differentiation. Similar patterns are observed in other endangered species with high mobility, such as the addax (*Addax nasomaculatus* [78]). Moreover, large admixed ancestral populations, such as the regent honeyeater, are expected to take more time to exhibit evidence of long ROH accumulation due to the extended coalescence times of their haplotypes [79]. This is evident from our genomic simulations, where larger ancestral populations show lower FROH values (figure 4c).

Our demographic reconstruction indicates a long-term decline from a large ancestral population in the regent honeyeater, beginning 2000 to 2500 years ago, with an accelerated decline in the last 500 years (figure 2*a*). This trend aligns with environmental changes that likely reduced suitable habitats. The early decline coincided with increased climate variability and intensified El Niño Southern Oscillation (ENSO) in Australia [80], which likely led to drier conditions and reduced eucalyptus forests—critical habitats for the regent honeyeater. In the past 70 years, there has been a sharp decline in effective population size (*N*_e_), consistent with declines attributed to habitat clearing of box-gum-ironbark woodlands [25,72,81,82] (figure 3). The long-term decline suggests that although the species has faced environmental pressures for an extended period, its historically large and geographically widespread and well-connected population may have helped buffer against genomic erosion. However, despite the expected accrued drift debt, the recent steep decline in *N*_e_ indicates that the species is now at a critical juncture. The current absence of significant inbreeding may not persist if population sizes remain low or continue to decrease, risking rapid loss of genetic diversity and increased inbreeding, jeopardizing long-term species viability.

### (b) Time lag of genetic diversity loss conceals the risk of population decline

The genome-wide heterozygosity of modern regent honeyeaters is higher than that of several other threatened species (figure 2*d*). Although the census population size has declined by more than 99%, the loss of genome-wide diversity was only 9%. This relatively high genetic diversity despite severe recent decline may result from the regent honeyeater’s historically large and widespread population. This finding aligns with the notion that global heterozygosity alone is not always a good predictor of conservation status [12,15].

A commonly overlooked factor is that genome-wide diversity loss and *N*_e_ reduction can be slow compared with census size decline (*N*_c_) [18,19,83]. This time lag is particularly acute in regent honeyeaters due to the species's large ancestral *N*_e_ and high mobility, which promote connectivity among populations and buffer diversity loss caused by genetic drift [18]. An *N*_e_ ≥ 500 has been recently adopted as an indicator in the Global Biodiversity Framework to preserve genetic diversity [84,85]. When direct estimates are not available, a common assumption is a 0.1 ratio between *N*_e_ and *N*_c_ at mutation-drift equilibrium [84–86]. However, population decline disrupts this equilibrium, and the slow rate of genetic diversity loss can inflate the *N*_e_/*N*_c_ ratio, masking true conservation risks [12,83,87]. For the regent honeyeater, the estimated long-term *N*_e_ is around >2 00 000 from estimates of nucleotide diversity (assuming a mutation rate of 4.6 × 10⁻⁹ [48]) and our more recent *N*_e_ estimate with GONE is approximately 13 000. Although we lack precise estimates of long-term *N*_c_, the ancestral population was likely very large, possibly in the hundreds of thousands. Following the collapse, the current *N*_c_ is approximately 250−300, resulting in a current *N*_e_/*N*_c_ ratio that significantly exceeds the assumed 0.1 ratio at mutation-drift equilibrium. Thus, for recently bottlenecked species, *N*_e_ alone may not be a reliable indicator of conservation concern. Therefore, accounting for both ancestral and recent demographic changes and their effects on the expected *N*_e_/*N*_c_ ratio is important when monitoring genetic diversity and assessing conservation status [12,83,85,87] (although robust estimates of *N*_e_ for large populations are challenging [88]).

The effect of the time lag of genomic erosion could be long-lasting as populations that experience strong bottlenecks accumulate a genetic drift debt [19]. Genetic diversity loss can occur long after the bottleneck started [19] and continue even despite demographic recovery [3]. This is of particular concern for declining species with large ancestral populations, which contain high level of masked genetic load [73], because the time-lag effect could conceal the signature of genomic erosion making predictions regarding fitness and population survival impacts challenging. Under severe population bottlenecks, drift could increase the frequency of deleterious variants before selection could purge them, potentially causing a decline in fitness [89]. Our simulations show that the portion of the genetic load expressed as negative fitness effects (realized load) peaks for the strongest bottlenecks and largest ancestral populations (figure 4*b*), placing the regent honeyeater at high risk for sustaining genomic erosion effects.

Overall, our results support that incorporating genomic diversity improves conservation assessments [5,16,83], and demonstrate how temporal genomic data can reveal hidden risks of genetic erosion missed by single-time-point metrics. These insights are crucial for species like the regent honeyeater, where genetic diversity remains deceptively high despite severe population declines.

### (c) Future environmental degradation will exacerbate extinction debt effects beyond genomic erosion risks

Regent honeyeaters face a genetic extinction debt, where despite current low inbreeding and moderate diversity loss, its persistence is immediately threatened by a rapidly degrading environment. Future environmental degradation could exacerbate extinction risks beyond genomic erosion for the regent honeyeater, emphasizing proactive conservation actions under an 'over-the-horizon' framework [90]. Our SDM predicts a significant decline in environmental suitability across the species current range, with future projections indicating severe range loss under all climate scenarios (figure 3). Breeding areas are predicted to suffer an even greater loss in suitability compared with the average loss across the study area, with 91% of the predicted loss happening before 2040. A decrease in suitable breeding grounds could exacerbate existing threats to the reproductive success of the regent honeyeater, like competition with other bird species for preferred breeding habitats and nest failure [91]. The species's reliance on group living makes it vulnerable to population decline due to the Allee effect, as its small, sparsely distributed population struggles to find nesting sites, forage efficiently, and defend resources [28,29,92]. These ecological challenges may lead to the species's rapid decline before genomic erosion significantly impacts fitness [28]. Therefore, the integration of species distribution models with temporal genomic data further highlights areas of ecological importance and provides a framework for predicting future at-risk habitats, emphasizing the need to consider both ecological and genomic factors in conservation planning.

### (d) Implications for conservation

We showcase the importance of considering the temporal genomic EBVs together with ecological insights in conservation assessments. Incorporating temporal genomic data enhances understanding of genomic erosion, complementing calls to integrate genomic diversity measures in conservation assessments [5,16,83]. For the regent honeyeater, the absence of significant inbreeding, coupled with the moderate loss of genetic diversity, underscores the importance of its continued monitoring. While the recent genetic diversity loss was modest, its long-term consequences and potential future inbreeding due to further declines should not be underestimated. Moreover, theory predicts that under population decline and drift debt, haplotypes and alleles will start to coalesce, increasing homozygosity and converting the substantial masked load of this species into a realized load [5,73]. Given that our sampling is 10 years old (approx. three generations), including more recent and future samples for genetic monitoring would be valuable.

Conservation strategies should aim to preserve genetic diversity and prevent inbreeding through habitat connectivity and, if necessary, genetic rescue efforts. Likewise, characterizing the genetic diversity in the existing captive populations to evaluate genetic rescue interventions could prove critical, particularly if both genetic diversity and genetic load are considered [93]. Overall, our results indicate that species monitoring must consider the trend of demographic decline [5], the baseline of genetic diversity [13], and the time lag of genetic diversity loss [18]. Moreover, while genetic factors are important, the ecological challenges facing the species are more concerning short-term and could lead to further declines and extinction if proper conservation strategies are not implemented [28].

## Data Availability

Sequencing reads have been deposited in the Electronic Research Data Archive (ERDA) at University of Copenhagen [94]. All the scripts to replicate the analyses have been deposited in Zenodo [95]. Supplementary material is available online [96].
